# Immunological efficacy of pneumococcal vaccine strategies in HIV-infected adults: a randomized clinical trial

**DOI:** 10.1038/srep32076

**Published:** 2016-09-01

**Authors:** C. Sadlier, S. O’Dea, K. Bennett, J. Dunne, N. Conlon, C. Bergin

**Affiliations:** 1 Department of GU Medicine and Infectious Diseases (GUIDE), St James’s Hospital, Dublin, Ireland; 2 School of Medicine, Trinity College Dublin, Dublin, Ireland; 3 Population Health Sciences Division, Royal College of Surgeons in Ireland, St Stephens Green, Dublin 2, Ireland; 4 Department of Immunology, St James’s Hospital, Dublin, Ireland

## Abstract

The aim of this study was to compare the immunologic response to a prime-boost immunization strategy combining the 13-valent conjugate pneumococcal vaccine (PCV13) with the 23-valent polysaccharide pneumococcal vaccine (PPSV23) versus the PPSV23 alone in HIV-infected adults. HIV-infected adults were randomized to receive PCV13 at week 0 followed by PPSV23 at week 4 (n = 31, prime-boost group) or PPSV23 alone at week 4 (n = 33, PPSV23-alone group). Serotype specific IgG geometric mean concentration (GMC) and functional oposonophagocytic (OPA) geometric mean titer (GMT) were compared for 12 pneumococcal serotypes shared by both vaccines at week 8 and week 28. The prime-boost vaccine group were more likely to achieve a ≥2-fold increase in IgG GMC and a GMC >1 ug/ml at week 8 (odds ratio (OR) 2.00, 95% confidence interval (CI) 1.46–2.74, p < 0.01) and week 28 (OR 1.95, 95% CI 1.40–2.70, p < 0.01). Similarly, the prime-boost vaccine group were more likely to achieve a ≥4-fold increase in GMT at week 8 (OR 1.71, 95% CI 1.22–2.39, p < 0.01) and week 28 (OR 1.6, 95% CI 1.15–2.3, p < 0.01). This study adds to evidence supporting current pneumococcal vaccination recommendations combining the conjugate and polysaccharide pneumococcal vaccines in the United States and Europe for HIV-infected individuals.

Invasive pneumococcal diseases (IPD) remains a significant cause of morbidity and mortality in HIV-infected individuals in the era of highly active antiretroviral therapy (HAART)^1^ (Harboe *et al*. 2014b). The incidence of IPD in HIV-infected individuals has been reported at up to 100 times greater than that of the general population^2^,^3^. In addition, HIV-infected individuals are at increased risk of recurrent episodes of IPD with up to one in four experiencing a recurrence during a 12 months period^4^,^5^.

The level of immunocompromise is considered the most important risk factor for IPD in HIV-infected individuals. Numerous studies have reported a decrease in incidence of IPD following the introduction of HAART, likely due to resultant immune-reconstitution^6^,^7^. However this has not been a consistent finding^8^,^9^,^10^.

PPSV23 has been shown to be safe and effective for the prevention of pneumococcal infection in the general adult population^11^ and covers the majority of pneumococcal serotypes implicated in IPD^12^,^13^. Given the burden of pneumococcal infection observed in HIV-infected individuals, PPSV23 has been recommended in consensus immunistation guidelines for HIV-infected adults in the US since 1996^14^,^15^. However, clinical and immunological efficacy of PPSV23 in HIV-infected adults remains debated^16^.

PCV13 has recently been added to immunization recommendations for HIV-infected adults although evidence supporting this recommendation for primary prevention of pneumococcal infection is limited^17^,^18^,^19^. The conjugate pneumococcal vaccine (PCV) has been shown to prevent pneumococcal pneumonia in HIV-infected children^20^ and to prevent recurrent IPD in HIV-infected adults^21^.

Immunogenicity studies with PCV, or combination regimens of PCV7 and PPSV23 conducted in HIV-infected adults have yielded variable results^22^,^23^,^24^,^25^,^26^,^27^,^28^,^29^.[Fig f1]

This study compares immunogenicity of a prime-boost immunization strategy combining the 13-valent conjugate pneumococcal vaccine (PCV13) at week 0 followed by the 23-valent polysaccharide pneumococcal vaccine (PPSV23) (prime-boost group) at week 4 versus PPSV23 at week 4 (PPSV23-alone group) in pneumococcal vaccine naïve HIV-infected adults.

The primary objective of this study was to compare serotype specific IgG geometric mean concentration (GMC) and opsonophagocytic (OPA) responses in the prime-boost versus the PPSV23-alone group for 12 serotypes (1, 3, 4, 5, 6B, 7F, 9V, 14, 18C, 19A, 19F, and 23F) contained in both vaccines at week 8 and week 28.

Secondary objectives were to compare the proportion of responses to the 12 shared pneumococcal polysaccharide serotypes (PPS) at week 8 and week 28 in the prime-boost versus the PPSV23-alone groups.

## Results

### Study Flow

Of 104 patients screened between April 2011 and July 2012, 64 patients (62%) were enrolled in the study. Thirty-one patients were randomized to receive PCV13 at week 0 followed by PPSV23 at week 4 (prime-boost group) and 33 were randomized to receive PPSV23 alone at week 4 (PPSV23-alone group).

60 patients completed the allocated vaccination series, 27 in the prime-boost group, 33 in the PPSV23 alone group.

In the prime-boost group two patients did not return within the time allocation to receive PPSV23, one patient withdrew consent and one patient was noted to have received PPSV23 prior to study entry and all were excluded from further analysis. Of 27 participants remaining in the prime-boost group, one patient did not attend for study bloods at week 8 and one patient did not attend for study bloods at week 28.

All 33 patients in the PPSV23-alone group received a single dose of PPSV23. Five patients did not attend for week 8 bloods and 6 patients did not attend for week 28 bloods.

### Patient Characteristics

Baseline characteristic of participants are outlined in Table 1. Mean age [SD] was 37 [9] years, 92% were male, mean CD4 count was 503 [209] cells/mm^3^. Prior to randomization, 47% of patients were on HAART. Mean HIV RNA was 4.5log_10_ copies/ml.

Patients in the prime-boost group were more likely to have a higher CD 4 T cell count (p = 0.03). No other significant differences in baseline characteristics were observed between groups.

### Serotype specific IgG response

At week 8, GMCs in the prime-boost group were higher versus the PPSV23-alone group for all serotypes with the exception of serotype 14. However, a statistically significant difference was reached for a single serotype only, 23F (3.20 vs. 0.52 *μ*g/mL, p < 0.01) (Table 2).

At week 28, GMC in the prime-boost group was significantly greater for 5 serotypes; 1 (0.48 vs 0.3 *μ*0g/ml, p = 0.05), 3 (1.21–0.56 g/m*μ*l, p = 0.02), 4 (0.17 vs. 0.42 *μ*g/ml, p = 0.02), 19F (1.51 vs. 0.88 *μ*g/ml, p = 0.04), 23F (1.54 vs. 0.42 *μ*g/ml., p = 0.013).

### Two-fold IgG serotype specific response

At week 8, number of participants in the PPSV23-alone group with a twofold increase in serotype specific IgG levels to 0, 1–3, 4–6, 7–9 and 10–12 pneumococcal polysaccharide serotypes (PPS) was 0, 2, 5, 9 and 12 versus 0, 1, 2, 4 and 19 in the prime-boost group. Twofold IgG response was observed more frequently in the prime-boost group versus the PPSV23 alone group (80% versus 70% respectively, OR 2.00, 95% CI 1.38–2.92, p < 0.01).

At week 28, the number of participants in the PPSV23-alone group with a two-fold increase in serotype specific IgG levels to 0, 1–3, 4–6, 7–9 and 10–12** **PPS was 0, 7, 8, 10 and 4 versus 0, 2, 4, 10 and 10 in the prime-boost group.

Twofold IgG response was observed more frequently in the prime-boost versus the PPSV23-alone group (70% versus 52% respectively, OR 2.19, 95% CI 1.59–3.03; p < 0.01).

### Two fold serotype specific IgG vaccine response and IgG > 1 ug/ml

A second more stringent criteria for vaccine response was applied where twofold increase in serotype specific IgG antibody levels and IgG concentrations >1 μg/ml was required.

Applying the more stringent criteria, at week 8 number of participants with two-fold IgG response and IgG >1 ug/ml to 0, 1–3, 4–6, 7–9 and 10–12 PPS were 0, 8, 8, 8, and 3 in the PPSV23-alone group versus 1, 3, 6, 6, and 10 in the prime-boost group. A greater frequency of response was observed in the prime-boost group versus the PPSV23-alone group (63% versus 46%, OR 2.00, 95% CI 1.46–2.74, p < 0.01).

At week 28, the frequency of responses to 0, 1–3, 4–6, 7–9 and 10–12 PPS were 2, 17, 5, 5, and 0 in the PPSV23-alone group versus 1, 7, 10, 6, and 2 in the prime-boost group. A greater frequency of response was observed in the prime-boost group versus the PPSV23-alone group (43% versus 27%, OR 1.95, 95% CI 1.40–2.70, p < 0.01).

### OPA GMT response

At week 8, OPA GMT was not significantly different in the prime-boost versus the PPSV23 alone group for 12 shared study serotypes. The greatest difference between serotypes observed was for serotype 23F where GMT was higher in the prime-boost group, 330.9 *μ*g/ml versus 31.56 *µ*g/ml in the unprimed group, although this was of borderline significance (p = 0.06) (Table 3).

At week 28, OPA GMT in the prime-boost group was significantly higher for serotype 14 (1010 versus 375.4 *μ*g/ml, p = 0.03) and serotype 23F (99.0 versus 18.3 *μ*g/ml, p = 0.03). GMT for serotype 6A (contained in PCV13 only) was significantly higher in the prime-boost group at week 8 (p < 0.01) and week 28 (p = 0.05).

At week 8 the proportion of serotypes with a 4-fold increase in GMT was 64% in the prime-boost group versus 51% in the PPSV23-alone group (OR 1.71, 95% CI 1.22–2.39, p < 0.01).

At week 28, the proportion of serotypes with a 4-fold increase in GMT remained greater in the prime-boost group 48% vs. 36% in the PPSV23-alone group (OR 1.6, 95% CI 1.15–2.3, p < 0.01).

## Discussion

Evidence supporting current Advisory Committee on Immunisation Practice (ACIP) and Infectious Diseases Society of America (IDSA) guidelines which recommend PCV13 followed by PPSV23 in HIV-infected individuals is limited^17^,^18^,^19^. This is the first study to investigate immunological response to PCV13 followed by PPSV23 versus PPSV23 in HIV-infected adults.

Studies evaluating antibody response to PPSV23 alone have consistently reported poor immunogenicity and lack of clinical efficacy has been observed in HIV-infected patients with CD4 cell counts <500/ml^21^,^30^,^31^.

We report a greater proportion of 2-fold IgG pneumococcal serotype responses in the prime-boost vaccine group compared to PPSV23-alone at week 8 and week 28 (OR 2.00, 95%CI 1.46–2.74 and 2.19, 95%CI 1.59–3.03 respectively). Frequency of serotype responses remained greater in the prime-boost group using the more stringent response definition (2-fold increase in IgG and IgG > 1 ug/ml) at week 8 and week 28 (OR 2.00, 95%CI 1.46–2.74 and 1.95, 95% CI 1.40–2.70 respectively).

Similarly, proportion of 4-fold serotype OPA responses was significantly greater in the prime-boost group versus the PPSV23-alone group at week 8 and week 28 (OR 1.71, 95%CI 1.22–2.39 and 1.6, 95% CI 1.15–2.3 respectively).

Our study findings support previously published research undertaken in HIV-infected adults by Lesprit *et al*. which reported a greater proportion of IgG and OPA serotype responses in the prime-boost immunization group versus PPSV23-alone group^22^.

IgG GMC was higher for all but one serotype in the prime-boost versus the PPSV23-alone group at week 8 however a statistically significant difference in GMC was not observed between groups. This may reflect limited power of the study due to lower than expected numbers being recruited. A study examining immunological response to a number of pneumococcal vaccine strategies, including a prime-boost immunization group similarly did not observe a significant increase in IgG GMC in the prime-boost group (n = 67)^24^.

In this study, IgG GMC declined more rapidly in the PPSV23-alone group versus the prime-boost vaccine group, and by week 28, IgG GMC was significantly greater in the prime-boost group for 5 serotypes (serotype 1, 3, 4, 19F, 23F) (Table 2). Other studies have reported a greater durability of antibodies with conjugate pneumococcal vaccine^32^.

We did not observe a significant difference in OPA GMT (widely regarded as the gold standard response measure for pneumococcal vaccine) at week 8 for the 12 shared pneumococcal serotypes between groups. At week 28 OPA GMT was significantly greater for 2 serotypes (14 and 23F).

Other studies investigating various response endpoints to pneumococcal vaccination in HIV-infected adults have reported variable immunological outcomes^23^,^24^,^25^,^26^,^27^,^28^,^29^.

Currently, there are no validated correlates of pneumococcal vaccine protection in adults. Historically, the most commonly accepted criteria for an adequate vaccination response to pneumococcal vaccine in adults have been either an absolute level of >1 μg/ml or a 4-fold increase in antibody concentration at a time point greater than 4 weeks following vaccination, usually in 70% of serotypes^33^,^34^.

Clinical data on the protective effects of these thresholds are limited, particularly in immunocompromised patient groups and as a result, a universal consensus does not exist. Lower thresholds have been suggested as correlates of response for the percentage of serotypes (50% versus 70%) and the fold change (2-fold versus 4-fold) required^35^,^36^ for a response to be documented.

Standardization of serological measures and reported responses, as well as further studies to identify immunologic correlates of protective response to pneumococcal vaccine in HIV-infected individuals are warranted.

Level of immunosuppression in HIV-infected individuals has been identified as one of the most important risk factors for IPD^6^,^7^. Low CD4 T cell count has also been shown to be associated with poor response to pneumococcal vaccine^31^,^37^,^38^. Immunization guidelines recommended pneumococcal vaccination with PCV13 irrespective of CD4 count as even an attenuated response will offer some protection in this high risk group.

Some studies suggest that individuals who are not on HAART or who have uncontrolled viraemia have diminished response to pneumococcal vaccine irrespective of CD4 count^39^. A recently undertaken randomized controlled trial assessing optimal timing of pneumococcal vaccine found that in HIV-infected individuals with CD4 count ≥200 cells/mm3, delaying PPSV23 until more than 6 months after initiating HAART did not improve responses and may lead to missed opportunities for immunization^40^.

We did not observe any adverse effects from vaccine during the course of the study. The safety of pneumococcal vaccine, both conjugate and polysaccharide has been demonstrated in other studies^39^,^40^.

## Limitations

Our study has several limitations. Sample size was small and more subjects withdrew or were lost to follow-up than expected making the final study group smaller than intended. We did not recruit sufficient numbers of participants to carry out a multivariate analysis thus we did not investigate differences in vaccine responses based on factors such as gender, smoking status, CD4 count, HIV viral load or HAART.

Baseline CD4 count in the prime-boost group was greater compared to the PPSV23 alone group which may represent a confounder. Duration of serological follow up (28 weeks) is relatively short and we cannot comment on sustainability of immunological response in the cohort. The majority of participants in this study were male Caucasians and thus results cannot be extrapolated to females or the wider population. In addition, whether the increase in antibody response observed for some serotypes after the conjugate vaccine in our study will translate into improved clinical efficacy is unknown.

## Conclusion

Our study suggests that combining PCV13 with PPSV23 elicits a greater magnitude of IgG and OPA immune response compared to PPSV23 alone in HIV-infected individuals with CD4 count >200 cells/mm^3^ over the study period.

Further studies addressing clinical end points and identifying immune correlates of vaccine protection are warranted as HIV-infected individuals will continue to be a group who will benefit greatly from protection against pneumococcal disease.

Our study adds to evidence supporting the current pneumococcal vaccination recommendations in the United States and Europe for HIV-infected individuals.

Ongoing monitoring of pneumococcal disease trends, particularly in high risk patient groups including those with HIV infection is warranted to determine the optimal pneumococcal prevention strategy in the future particularly as extended valency vaccines become available.

## Methods

This study was undertaken in an ambulatory HIV clinic in the Department of Genitourinary Medicine and Infectious Diseases (GUIDE), St James’s Hospital, Dublin, Ireland.

Pneumococcal vaccine naïve HIV-infected individuals >18 years with CD4 cell counts ≥200 cells/mm^3^ meeting eligibility criteria outlined in the study protocol (EudraCT number 2011-000260-99) were recruited between April 2011 and July 2012. As international pneumococcal vaccine recommendations changed in 2012 to recommend PCV13 followed by PPSV23 for all HIV-infected adults recruitment to this study was discontinued and the prime boost immunisation strategy implemented as standard of care for all patients attending our clinic.

Participants were randomized by computer generated allocation sequence with equal probability of being randomized to PCV13 (Prevnar; Wyeth Pharmaceuticals) at week 0 followed by PPSV23 at week 4 (prime boost group) or PPSV23 alone (Pneumovax; Merck) at week 4 (PPSV23 group). All subjects provided written informed consent.

Vaccines were supplied in pre-filled single-dose syringes without preservatives and stored according to manufacturer guidelines at 2–8 °C. Vaccines were injected intramuscularly (0.5 mL) in the deltoid muscle with the use of a 23-gauge, 1-inch needle.

## Study Objectives

The primary objective of this study was to compare serotype specific IgG geometric mean concentration (GMC) and opsonophagocytic (OPA) responses in the prime-boost versus the PPSV23-alone group for 12 serotypes (1, 3, 4, 5, 6B, 7F, 9V, 14, 18C, 19A, 19F, and 23F) contained in both vaccines at week 8 and week 28.

Secondary objectives were to compare the proportion of responses to the 12 shared pneumococcal polysaccharide serotypes (PPS) at week 8 and week 28 in the prime-boost versus the PPSV23-alone groups.

## Immunogenicity

### IgG geometric mean concentration

Serum concentrations of anti-capsular IgG for 12 shared PPS (1, 3, 4, 5, 6B, 7F, 9V, 14, 18C, 19A, 19F, and 23F) were determined using a validated enzyme-linked immunosorbent assay and expressed as micrograms per milliliter (μg/ml)^41^.

Briefly, test sera were pre-adsorbed with C-PS (5 *μ*g/ml) to remove nonfunctional antibodies. A further adsorption step was included with serotype 22F polysaccharide (5 *μ*g/ml). The lower limit of detection is 0·01 *μ*g/ml^41^.

### Opsonophagocytic geometric mean titer

Opsonophagocytic assays measure complement mediated killing, one of the main protective responses against *S. pneumoniae* infection and are widely accepted as the gold standard measure of pneumococcal vaccine response. Functional antibacterial OPA activity was measured using 13 serotype-specific validated assays and comparisons made for the 12 serotypes contained in both vaccines.

Briefly, heat-inactivated sera were serially diluted 2.5-fold in buffer. Target bacteria were added to assay plates and were incubated for 30 min at 25 °C on a shaker. Baby rabbit complement (3–4 week old, Pel-Freez, 12.5% final concentration) and differentiated HL-60 cells, were then added to each well at an approximate effector to target ratio of 200:1. Assay plates were incubated for 45 min at 37 °C on a shaker. To terminate the reaction, 80 μL of 0.9% NaCl was added to all wells, mixed, and a 10-μL aliquot were transferred to the wells of Millipore, MultiScreenHTS HV filter plates containing 200 μL of water. Liquid was filtered through the plates under vacuum, and 150 μL of HySoy medium was added to each well and filtered through. The filter plates were then incubated at 37 °C, 5% CO_2_ overnight and were then fixed with Destain Solution (Bio-Rad). The plates were then stained with Coomassie Blue and destained once. Colonies were imaged and enumerated on a Cellular Technology Limited (CTL) ImmunoSpot Analyzer^®^. The OPA antibody titer was interpolated from the reciprocal of the two serum dilutions encompassing the point of 50% reduction in the number of bacterial colonies when compared to the control wells that did not contain immune serum^42^,^43^.

IgG concentrations and opsonophagocytic titers were measured in blood samples obtained before (week 0) vaccination, at week 8 and week 28 post vaccination.

The laboratory measurements were performed blinded to case-control status.

### Definitions and statistical methods

In the first instance serotype specific IgG response was defined as a 2-fold increase from baseline of serotype specific IgG antibody levels (μg/ml).

A second more stringent definition of response was applied. This was defined as a 2-fold increase in serotype specific IgG and an IgG levels >1 μg/ml.

OPA response was defined as a 4-fold or greater increase in OPA titer.

Descriptive statistics are presented as *N* (percentages) for categorical values, means with standard deviations (SDs) for numerical values.

IgG concentrations (reported as *μ*g/ml) and OPA titers (reported as *μ*g/ml) are expressed as geometric means with 95% CIs using logarithmically transformed assay results.

The Wilcoxon or Mann-Whitney U test were used compare pre and post vaccination IgG and OPA responses between groups study.

Proportion of vaccine serotype responses between vaccine groups was compared using the chi square test.

Data analyses were performed using Graphpad prism (version 6) and SPSS (version 22). No imputations were performed for missing data.

### Ethical considerations

This was a single center study undertaken in an ambulatory HIV out-patient’s clinic in the Department of Genitourinary Medicine and Infectious Diseases (GUIDE), St James’s Hospital, Dublin, Ireland from April 2011-July 2012. The study protocol was approved by the St James’s Hospital/Tallaght Hospital Research Ethics Committee (approval number 10102010) and the Irish Medicines Board (approval number 2095901). This study was registered on the European Clinical Trials Database on 04/12/2011 (EudraCT number 2011-000260-99). Written informed consent was obtained from all study participants. All study methods were performed in accordance with the relevant guidelines and regulations.

## Additional Information

**How to cite this article**: Sadlier, C. *et al*. Immunological efficacy of pneumococcal vaccine strategies in HIV-infected adults: a randomized clinical trial. *Sci. Rep.* **6**, 32076; doi: 10.1038/srep32076 (2016).

## Figures and Tables

**Figure 1 f1:**
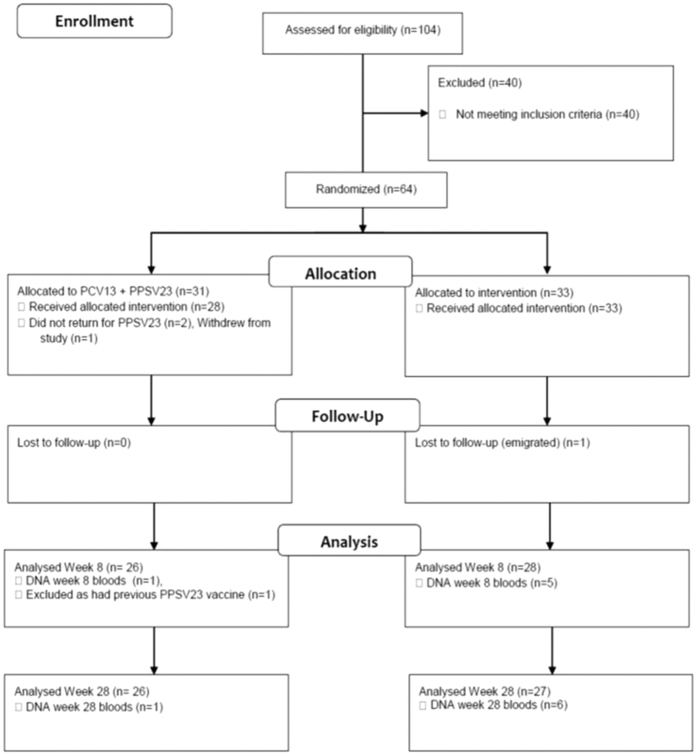
Study flow of vaccination with PCV13 and PPSV23 or PPSV23 alone and follow up.

**Table 1:** Baseline characteristics of patients by vaccine group.

	TOTAL (n = 60)	PPSV 23 group (n = 33)	PCV13 + PPSV23 (n = 27)	P
Male n, (%)	55 (92)	29 (88)	26 (96)	0.37
Age (mean) [SD]	37 [10]	37 [10]	36 [10]	0.70
Race n, (%)				
Caucasian	48 (80)	25 (76)	23 (85)	0.52
Hispanic	7 (12)	4 (12)	3 (11)	1
Black	5 (8)	4 (12)	1 (4)	0.37
Risk of Acquisitions n, (%)				
MSM	49 (82)	25 (76)	24 (89)	0.32
Hetero	8 (13)	6 (18)	2 (7)	0.28
IDU	3 (5)	2 (6)	1 (4)	0.49
CD4 + count (mean) [SD]	503 [219]	447 [181]	572 [243]	0.03
On HAART n, (%)	28 (47)	17 (52)	11 (40)	0.09
HIV log_10_ (mean) [SD]	2.37 [2.1]	2.16 [2]	3 [2]	0.45
Smoker n, (%)	20 (33)	11 (33)	9 (27)	1.1
Co-infection Hepatitis C (PCR positive)	5	3	2	1.0

Abbreviations: HAART, highly active antiretroviral therapy; SD, standard deviation; MSM, Men who have sex with men; Hetero, Heterosexual, IDU, intravenous drug user.

**Table 2:** Geometric mean antibody concentration (μg/mL) for polysaccharide pneumococcal serotypes.

Serotype	W 0 IgG GMC^b^ (95% CI)		p^a^	W 8 IgG GMC^b^ (95% CI)		p^a^	W 28 IgG GMC^b^ (95% CI)		p^a^
	PCV13 + PPSV23 n = 27	PPSV23 n = 33		PCV13 + PPSV23 n = 26	PPSV23 n = 28		PCV13 + PPSV23 n = 26	PPSV23 n = 27	
1	0.17 (0.05–0.37)	0.14 (0.09–0.19)	0.59	0.86 (0.51–1.94)	0.73 (0.45–1.17)	0.45	0.48 (0.25–0.77)	0.30 (0.21–0.43)	0.05
3	0.38 (0.11–1.07)	0.47 (0.26–0.87)	0.77	2.16 (1.21–3.42)	1.18 (0.60–2.30)	0.06	1.21 (0.84–1.98)	0.56 (0.33–0.95)	0.02
4	0.12 (0.05–0.15)	0.11 (0.08–0.15)	0.77	0.82 (0.33–1.26)	0.35 (0.19–0.64)	0.08	0.41 (0.23–0.57)	0.17 (0.12–0.24)	0.01
5	0.18 (0.05–0.40)	0.15 (0.10–0.24)	0.77	1.68 (0.84–4.77)	1.01 (0.49–2.09)	0.35	0.83 (0.44–1.97)	0.42 (0.21–0.81)	0.09
6B	0.18 (0.05–0.44)	0.18 (0.11–0.27)	0.84	1.56 (0.84–3.67)	0.81 (0.33–1.99)	0.20	0.82 (0.33–2.03)	0.49 (0.25–0.96)	0.18
7F	0.51 (0.24–0.29)	0.49 (0.33–0.71)	0.61	1.97 (0.80–4.38)	2.14 (1.29–3.54)	0.89	1.34 (0.72–2.19)	1.21 (0.80–1.86)	0.77
9V	0.29 (0.16–0.63)	0.27 (0.17–0.44)	0.7524	1.97 (0.90–4.38)	1.17 (0.63–2.18)	0.19	0.99 (0.52–2.09)	0.70 (0.38–1.25)	0.44
14	0.44 (0.16–0.63)	1.04 (0.57–1.90)	0.07	4.61 (1.88–20.96)	5.71 (3.15–10.36)	0.93	2.91 (1.24–8.15)	3.44 (2.03–5.81)	0.91
18C	0.17 (0.05–0.27)	0.32 (0.20–0.52)	0.06	3.67 (0.85–13.36)	1.81 (0.98–3.36)	0.20	1.44 (0.42–5.39)	1.08 (0.58–2.01)	0.73
19A	0.52 (0.32–0.27)	0.66 (0.40–1.10)	0.61	3.69 (1.65–10.80)	3.48 (1.63–7.40)	0.72	1.94 (0.84–4.11)	2.11 (1.10–4.02)	0.92
19F	0.43 (0.24–0.64)	0.45 (0.28–0.71)	0.45	2.90 (1.64–4.7)	1.54 (0.86–2.76)	0.18	1.51 (1.05–1.99)	0.88 (0.57–1.36)	0.04
23F	0.25 (0.05–0.92)	0.17 (0.11–0.28)	0.45	3.20 (0.86–14.44)	0.55 (0.28–1.07)	<0.01	1.55 (0.63–4.78)	0.42 (0.24–0.75)	0.01

Abbreviations: W, week; PCV13, 13-valent pneumococcal conjugate vaccine; PPSV23, 23-valent pneumococcal polysaccharide vaccine, n, number of patients with valid assay results for the specified serotype.

^a^Student test or Wilcoxon test.

^b^GMCs were calculated using all subjects with available data for the specified blood collection. GMCs expressed as micrograms per millilitre (μg/mL).

**Table 3:** Geometric mean antibody titres (ug/ml) for pneumococcal OPA.

Serotype	W0 GMT (95% CI)		p^a^	Week 8 GMT (95% CI)		p^a^	Week 28 GMT (95% CI)		p^a^
	PCV13 + PPSV23 n = 27	PPSV23 n = 33		PCV13 + PPSV23 n = 26	PPSV23 n = 28		PCV13 + PPSV23 n = 26	PPSV23 n = 27	
1	4.98 (4.07–6.08)	4.80 (4.02–5.73)	0.89	27.35 (13.97–53.54)	33.42 (18.11–61.66)	0.85	12.20 (7.31–20.37)	12.34 (8.51–17.91)	0.29
3	5.28 (3.63–7.69)	5.28 (3.80–7.32)	0.91	19.53 (11.78–32.37)	13.36 (7.46–23.93)	0.66	6.91 (4.50–10.61)	5.95 (4.33–8.18)	0.36
4	10.19 (4.48–23.17)	6.77 (3.66–12.53)	0.67	938 (397–2216)	431 (153–1218)	0.26	281 (95.08–828)	50.05 (15.96–157)	0.26
5	5.39 (4.10–7.09)	4.42 (3.79–5.16)	0.24	55.19 (27.21–112)	24.60 (12.12–49.93)	0.80	22.02 (11.73–41.34)	10.36 (5.94–18.09)	0.19
6A	8.14 (3.98–16.66)	9.89 (4.83–20.25)	0.66	618 (238–1607)	38.55 (13.44–111)	<0.01	209 (72.90–598)	23.02 (7.95–66.66)	0.05
6B	68.12 (16.63–279)	81.54 (22.04–302)	0.82	738 (250–2178)	717 (253–2028)	0.40	522 (184–1479)	643 (266–1553)	0.68
7F	11.87 (5.10–27.64)	10.19 (4.97–20.91)	0.81	1354 (809–2267)	613 (295–1277)	0.21	395 (191–816)	142 (47.69–424)	0.88
9V	94.74 (26.67–337)	73.21 (24.02–223)	0.66	1324 (701–2502)	955 (386–2363)	0.65	1193 (772–1844)	292 (95.85–892)	0.31
14	190 (57.69–624.9)	414 (222–773)	0.24	2544 (1721–3762)	1266 (827–1938)	0.11	1010 (541–1886)	375 (185 762)	0.03
18C	13.69 (4.84–38.74)	7.92 (3.87–16.21)	0.81	1090 (466–2551)	295 (108–806)	0.23	225 (78.75–645)	97.45(34.98272)	0.20
19A	8.84 (5.44–14.38)	17.64 (9.78–31.81)	0.30	239 (127–452)	228 (127–408)	0.55	88.37 (48.72–160)	85.80 (46.80–157)	0.76
19F	10.41 (6.22–17.42)	13.24 (7.12–24.60)	0.31	274 (134–560)	157 (80.43–305)	0.47	57.10 (30.79–106)	30.66 (16.42–57.25)	0.50
23F	7.68 (3.90–15.10)	7.16 (4.03–12.74)	0.81	331 (128–856)	31.65 (11.79–85.00)	0.06	99.02 (37.64–261)	18.32 (8.50–39.52)	0.03

Abbreviations: W, week; PCV 13, 13-valent pneumococcal conjugate vaccine; PPSV23, 23-valent pneumococcal polysaccharide vaccine; number of patients with valid assay results for the specified serotype.

^a^Student test or Wilcoxon test b GMTs were calculated using all subjects with available data for the specified blood collection.
